# Fecal Short-Chain Fatty Acids to Predict Prediabetes and Type 2 Diabetes Risk: An Exploratory Cross-Sectional Study

**DOI:** 10.3390/nu17183003

**Published:** 2025-09-19

**Authors:** Rocío Puig, Marina Idalia Rojo-López, Josep Julve, Esmeralda Castelblanco, Julia Ponomarenko, Susana Amézqueta, Joan Vendrell, Josep Franch-Nadal, Josep Lluís Torres, Dídac Mauricio, Sara Ramos-Romero

**Affiliations:** 1 Department of Endocrinology and Nutrition, Hospital de la Santa Creu i Sant Pau, 08041 Barcelona, Spain; rpuig@santpau.cat; 2 Group of Endocrinology, Diabetes & Nutrition, Institut de Recerca Sant Pau (IR Sant Pau), Sant Quintí 77-79, 08041 Barcelona, Spain; nut.marina.rojo.l@gmail.com (M.I.R.-L.); jjulve@santpau.cat (J.J.); 3 Departament of Medicine, Universitat Autònoma de Barcelona, 08035 Barcelona, Spain; 4 DAP-Cat Group, Unitat de Suport a la Recerca Barcelona, Fundació Institut Universitari per a la Recerca a l’Atenció Primària de Salut Jordi Gol i Gurina (IDIAPJGol), 08007 Barcelona, Spain; 5 Centro de Investigación Biomédica en Red de Diabetes y Enfermedades Metabólicas Asociadas (CIBERDEM), Instituto de Salud Carlos III (ISCIII), 08041 Barcelona, Spain; juanjose.vendrell@urv.cat (J.V.); josep.franch@gmail.com (J.F.-N.); 6 Endocrinology, Metabolism and Lipid Research Division, Department of Medicine, Washington University School of Medicine, St. Louis, MO 63110, USA; esmeraldacas@gmail.com; 7 Centre for Genomic Regulation, The Barcelona Institute of Science and Technology, Universitat Pompeu Fabra (UPF), 08003 Barcelona, Spain; julia.ponomarenko@crg.eu; 8 Departament d’Enginyeria Química i Química Analítica, Institut de Biomedicina (IBUB), Universitat de Barcelona, 08028 Barcelona, Spain; samezqueta@ub.edu; 9 Departament of Endocrinology and Nutrition, Hospital Universitari Joan XXIII de Tarragona, Institut d’Investigació Sanitària Pere Virgili (IISPV), 43005 Tarragona, Spain; 10 Medicine School, Universitat Rovira i Virgili (URV), 43204 Reus, Spain; 11 Primary Health Care Center Raval Sud, Gerència d’Atenció Primaria, Institut Català de la Salut, 08001 Barcelona, Spain; 12 Department of Biological Chemistry, Institute of Advanced Chemistry of Catalonia (IQAC-CSIC), 08034 Barcelona, Spain; joseplluis.torres@iqac.csic.es; 13 Nutrition & Food Safety Research Institute (INSA-UB), Maria de Maeztu Unit of Excellence, 08921 Santa Coloma de Gramenet, Spain; 14 Faculty of Medicine, University of Vic—Central University of Catalonia, 08500 Vic, Spain; 15 Department of Cell Biology, Physiology and Immunology, Faculty of Biology, University of Barcelona, 08028 Barcelona, Spain

**Keywords:** dysglycemia, dietary intake, clinical studies, microbial metabolites, gut microbiota, SCFA profiles, metabolic risk factors

## Abstract

**Background/Objectives:** Gut microbiota is profoundly influenced by dysglycemic states, including prediabetes (preDM) and type 2 diabetes (T2D). Although short-chain fatty acids (SCFAs) may serve as proxies reflecting these microbial changes, their predictive role remains elusive. This study aimed to evaluate the association between fecal SCFA concentrations and glycemic status (preDM and T2D), using individuals with normoglycemia (NonDM) as the reference group in a Mediterranean adult population. **Methods:** This study included a total of 88 participants from the Di@bet.es study who were classified into three groups according to the American Diabetes Association criteria: NonDM (45%), preDM (27%), and T2D (28%), respectively. We evaluated gut microbiota populations through massive sequencing and determined SCFAs concentration using gas chromatography–mass spectrometry. Adjusted multiple logistic regression models were used to estimate associations between SCFAs and metabolic status. **Results**: The mean age of subjects with preDM and T2D was approximately 68 years, older than that of NonDM participants (about 60 years). About 50% of the subjects in the NonDM and preDM groups were female, whereas in the T2D group, females represented about 25%. The analysis revealed that only fecal acetic acid was significantly reduced in T2D compared to NonDM (*p* = 0.036) and preDM (*p* = 0.018) groups. Remarkably, fecal acetic acid was negatively associated with T2D risk when taking preDM as the reference state (OR = 0.561 [95% CI: 0.371–0.846], *p* = 0.009). Intriguingly, fecal acetic acid was identified as a significant positive predictor of preDM risk, taking the NonDM group as reference (OR = 1.422; *p* = 0.028), while propionic acid was inversely associated with preDM (OR = 0.714; *p* = 0.028). **Conclusions**: Our analysis showed that fecal acetic acid levels were associated with a reduced risk of T2D but also with an increased risk of preDM; however, the biological relevance of these findings remains uncertain.

## 1. Introduction

The worldwide incidence of prediabetes (preDM), recognized as the preclinical phase of type 2 diabetes (T2D), has shown a steady increase over time, representing a substantial public health challenge. The occurrence of preDM is contingent upon the demographic characteristics of the studied cohort as well as the diagnostic criteria employed [1]. The International Diabetes Federation (IDF) projected that in the year 2021, approximately 541 million individuals globally were afflicted with impaired glucose tolerance [2]. Roughly nine out of ten diabetes diagnoses correspond to T2D [3].

PreDM and T2D result from a complex interplay of modifiable and non-modifiable individual, social, and environmental factors [4]. Understanding these factors and their interplay is crucial for developing effective prevention and management strategies. Both conditions have a strong genetic component and are closely associated with obesity and a sedentary lifestyle. A dietary pattern characteristic of Western populations—high in refined carbohydrates, red/processed meats, and added sugars—has been consistently linked to higher risk of preDM and T2D [5]. Conversely, healthier dietary patterns, such as those rich in vegetables, fruits, and whole grains, have been shown to reduce the incidence of preDM, and therefore the risk of T2D [6]. Genetic factors also influence the relationship between diet and T2D risk, making individuals with specific genetic backgrounds more susceptible to the adverse effects of unhealthy dietary patterns [7]. Moreover, the relationship between insulin resistance and obesity is well recognized, with central obesity acting as a key driver in the onset of metabolic dysfunction-associated steatotic liver disease (MASLD), a disorder commonly seen in preDM and T2D [8].

Emerging evidence highlights the key role of gut microbiota (GM) in regulating energy balance and metabolism through compounds like short-chain fatty acids (SCFAs), which interact with host tissues [9]. SCFAs play a key role in energy and lipid metabolism, with research showing that butyric supplementation in high-fat diets reduces obesity and insulin resistance in experimental animals [10]; however, these results cannot be directly extrapolated to humans [11]. Moreover, alterations in gut microbial composition have been linked to the development of obesity and T2D, primarily through mechanisms involving inflammation, metabolic endotoxemia, and disrupted nutrient metabolism [12]. Studies have observed reduced gut microbial diversity in individuals with prediabetes and newly diagnosed T2D compared to those without diabetes [13]. However, knowledge about gut dysbiosis in the early stages of glucose intolerance remains limited. Previous research has shown variations in the gut microbiome of preDM and individuals with established T2D compared to healthy individuals, with distinct microbial profiles associated with the disease [13]. Furthermore, longitudinal and cross-sectional studies have demonstrated a complex, stage-dependent interaction between the GM and the host during the progression from prediabetes to type 2 diabetes [14,15]. In this context, the Mediterranean dietary pattern has been associated with higher fecal SCFA concentrations and lower levels of intestinal permeability markers, suggesting a clear mechanistic link between SCFAs, intestinal barrier integrity, and diet [16].

Clarifying the interplay between gut microbial composition, SCFA production, and the development of prediabetes and type 2 diabetes could provide key insights into disease mechanisms. We hypothesized that distinct fecal SCFA patterns are associated with glycemic status and reflect underlying alterations in gut microbiota composition. Therefore, this study aimed to evaluate the associations between gut microbiota composition, fecal SCFAs, and glycemic status (normoglycemia, preDM, and T2D) in adults from a Mediterranean population.

## 2. Materials and Methods

### 2.1. Study Design and Subjects

This work is a cross-sectional sub-study of the Di@bet.es cohort, a population-based study conducted in Spain to investigate the epidemiology and risk factors of diabetes [17].

For the present study, 88 individuals from the Mediterranean region participating in the Di@bet.es study were selected based on the availability of complete clinical data and fecal, urine, and plasma samples [18]. Participants were distributed into three groups according to glycemic status: NonDM (*n* = 39, 45%), preDM (*n* = 24, 27%), and T2D (*n* = 25, 28%). The mean age of participants with preDM and T2D was approximately 68 years, higher than that of NonDM (~60 years). About half of the NonDM and preDM groups were female, whereas only 25% of the T2D group were women. Exclusion criteria, as defined in the original Di@bet.es study, included being institutionalized, severe illness, pregnancy, or recent delivery. The study was approved by the Ethics and Clinical Research Committee of IDIAP Jordi Gol (P17/085, 26 April 2017), with written informed consent acquired from all individuals involved.

Urine and fecal samples from participants were frozen at −80 °C until analysis and thawed on the day of processing.

### 2.2. Study Procedures

Clinical and biochemical variables, including serum lipid profile, waist circumference, body mass index (BMI), and other anthropometric measures, were obtained following the methodology of the Di@bet.es study, as previously described [17,18].

### 2.3. Classification of Glycemic Status

Participants were categorized as NonDM, preDM, or T2D based on American Diabetes Association (ADA) diagnostic thresholds [19].

### 2.4. Food Frequency Questionnaire

A qualified nutritionist administered a qualitative food frequency questionnaire (FFQ), which had been previously employed in a population-based investigation conducted in Spain [20]. The nutritionists were informed of the participants’ prior diabetic condition. The FFQ was facilitated face-to-face, and participants provided a single response for each food item indicating the frequency of consumption. The questionnaire assessed the annual frequency of consumption of 50 food items categorized into 11 groups: never/seldom, 1 and 2–3 times/month, 1, 2–3, and 4–6 times/week, and 1, 2, 3, 4, and >4 times/day. For the present analysis, food items were grouped into the following categories: vegetables, fruit, dairy, potatoes, legumes, nuts, non-refined cereals, fish, red meat, white meat, wine, beer, coffee, and sweeteners.

### 2.5. Urinary Sugar Excretion Analysis

Sucrose, glucose, and fructose were quantified in first-morning urine using a commercial enzymatic assay (Saccharose/D-Glucose/D-Fructose kit; Boehringer Mannheim, R-Biopharm, Darmstadt, Germany; No. 10716260035) and measured with a UV-VIS spectrophotometer (SP8001, Dinko Instruments, Barcelona, Spain). On the assay day, urine samples were homogenized and centrifuged (15,000 rpm, 4 °C, 4 min) prior to measurement. Only those samples with glucose concentrations above the detection range of the enzymatic kit were diluted 1/10 (*v*/*v*) with water.

Glucose, creatinine, and albumin measures were further determined using commercial kits (Roche Diagnostics) adapted to a COBAS c501 autoanalyzer (Roche Diagnostics Cobas 6000 c501 GmbH, Mannheim, Germany) for verification purposes.

### 2.6. Fecal DNA Extraction and Sequencing for Microbiota Populations Analysis

Extraction of total DNA from fecal material was performed with the QIAamp™ DNA Stool Mini Kit (QIAGEN, Hilden, Germany). The V3–V4 segments of the 16S rRNA gene were subsequently amplified and sequenced using Illumina MiSeq technology (paired-end 2 × 300 bp). Bioinformatic analysis of sequencing data was performed using the mothur software (v1.44.1) [21], with taxonomic classification based on the SILVA v132 database [22] and clustering at the genus level into operational taxonomic units (OTUs). Chimeric and non-bacterial sequences were removed prior to statistical analysis. Further methodological details are provided in the Supplementary Methods.

### 2.7. Fecal Short-Chain Fatty Acids

SCFAs in stool were quantified with gas chromatography using a Trace2000 system coupled to a flame ionization detector (ThermoFinnigan, Waltham, MA, USA) and an HP-FFAP capillary column (30 m × 0.53 mm × 1 μm; Agilent, Santa Clara, CA, USA). Helium was employed as carrier gas at 5 mL/min. The oven temperature program started at 80 °C (1 min), then increased to 120 °C at 15 °C/min (4 min), followed by 130 °C at 5 °C/min (4 min), and finally to 235 °C at 8 °C/min (4 min). The final detector temperature was 240 °C. External calibration was performed for six SCFAs (acetate, propionate, butyrate, isobutyrate, valerate, isovalerate) using ethylbutyric acid as internal standard. Extraction from feces was performed with acetonitrile/water/oxalic acid, and quantification was based on the respective calibration curves. Total SCFA concentration was obtained as the sum of individual acids.

### 2.8. Statistical Analyses

All analyses were performed using R software (version 4.3.1), with data cleaning, management, and visualization conducted in an R Markdown notebook to ensure full traceability and reproducibility. The packages dplyr, tidyr, and purrr were used for data manipulation; mice for imputing missing values; compareGroups for group comparisons; broom and FSA for inferential statistics; and ggplot2 and kableExtra for tables and plots.

Categorical variables were tested using Chi-square tests, and continuous variables with Wilcoxon or Kruskal–Wallis tests when non-normal distributions were present. Results are reported as mean ± SD or median with interquartile range.

The 16S rRNA OTUs counts were analyzed using the R package Phyloseq (version 1.36.0) [23] in R version 4.1.0. Statistical significance of the relative abundances of taxa (>0.01) was evaluated using the Wald test of the DESeq2, version 1.32.0. [24]. In addition, the R package microbiome (version 1.13.12) [25] was used to calculate indexes to estimate within-sample α-diversity, which is the distribution of taxonomic units’ relative abundances in a given sample into a single number, including those for richness (i.e., Chao1 index), evenness (i.e., Pielou, Simpson, and Bulla indexes), dominance (i.e., Simpson, Berger–Parker, Relative, and Gini indexes), rarity (i.e., low abundance index), and diversity (i.e., Shannon and Inverse Simpson indexes). Furthermore, representation of similarities or distances between samples, referred to as between-sample β-diversity (i.e., principal coordinate analysis of unweighted and weighted Unifrac distance), and its significance to differentiate groups (Permanova analysis with Adonis) were calculated using the R packages Phyloseq and vegan (version 2.5.7) [26].

Multiple logistic regression models (glm, family = “binomial”) were used to estimate associations between independent variables (e.g., dietary patterns, clinical markers, bacterial composition) and metabolic status, adjusting for confounders. Results are presented as odds ratios (ORs) with 95% confidence intervals, using *broom* for output.

Correlations between bacterial taxa and clinical or dietary factors were assessed with Spearman coefficients. Volcano plots (ggplot2) were generated to visualize correlation strength and statistical significance (−log10(*p*-value)).

Several figures, including plots of the logistic regression models, correlation heatmaps, and relative abundance barplots, were generated using Python in Google Colab (matplotlib 3.8.0, seaborn 0.12.2), ensuring reproducibility through a shared Python notebook.

## 3. Results

### 3.1. Demographic and Clinical Characteristics

The clinical and metabolic characteristics of subjects included in the study are shown in Table 1.

The waist circumference was significantly higher only in the group of subjects with T2D when compared with NonDM (*p* < 0.01). Both glucose and HbA1c were increasingly higher across the groups of subjects with preDM and T2D and were significantly elevated compared with NonDM subjects. Lipid analysis revealed that total cholesterol was significantly reduced in the group of subjects with T2D compared with the preDM group and, though marginally, with the NonDM group. The latter was mainly due to concomitant reductions in the low-density lipoprotein (LDL) and high-density lipoprotein (HDL) cholesterol in the T2D group compared with the other two groups. Total triglycerides were marginally elevated in the preDM and T2D groups compared with the NonDM group. As expected, urinary glucose was higher in individuals with T2D (2.31 ± 6.21 g/L) compared to those with preDM (0.08 ± 0.05 g/L) and NonDM (0.45 ± 1.42 g/L), with a *p*-value close to significance (*p* = 0.051). Fructose and sucrose levels were also higher in T2D, although differences did not reach statistical significance.

### 3.2. Dietary Intake Patterns

The frequency of intake of fiber-rich food groups (e.g., vegetables, legumes, whole grains), fish, read meat or nuts did not differ among groups (Supplementary Table S1). By contrast, some food groups and beverages showed significant differences across glycemic categories (Table 2).

The complete dietary dataset, including variables without significant differences, is available in Supplementary Table S1.

Dairy consumption was higher in individuals with preDM compared to NonDM subjects (*p* = 0.040). Intake of white meat was significantly higher in individuals with T2D compared to those with preDM (*p* = 0.027) and marginally higher compared to NonDM (*p* = 0.055). Among beverages, coffee consumption was significantly higher in the preDM group compared to NonDM (*p* = 0.010) and approached statistical significance between preDM and T2D (*p* = 0.056). Beer intake was lower in individuals with preDM and T2D compared to NonDM (*p* = 0.047), with a significant difference between NonDM and preDM (*p* = 0.019).

### 3.3. Composition of Gut Microbiota in Feces

Microbial alpha diversity did not differ significantly between glycemic status groups (χ^2^ = 1.02, *p* = 0.60). Microbial beta diversity analysis showed a marginally significant difference in overall community composition across groups (R^2^ = 0.033, *p* = 0.05). The relative abundance of the main bacterial phyla—Bacteroidetes, Firmicutes, Proteobacteria, and others—across glycemic groups (NonDM, preDM, and T2D) is summarized in Supplementary Figure S1 and detailed in Supplementary Table S2.

At the phylum level, Firmicutes were predominant in all groups, representing over 75% of the total. No statistically significant differences were found between groups for any phylum.

**Figure 1:**
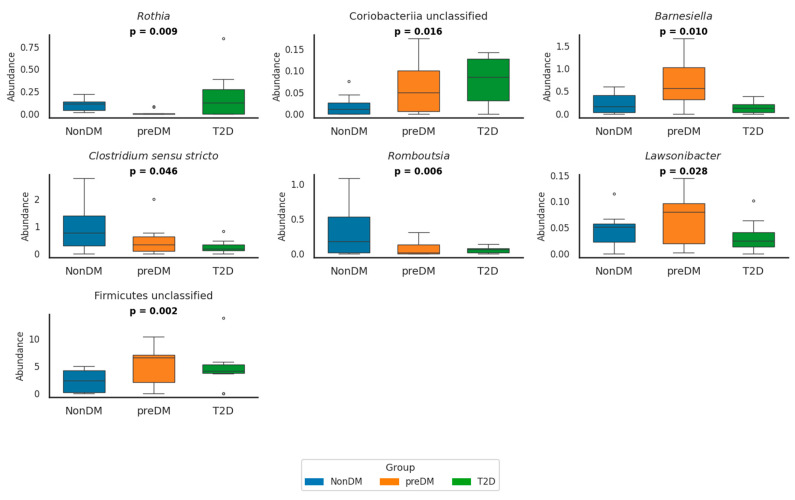
Relative abundance of seven bacterial genera in NonDM (blue), preDM (orange), and T2D (green) groups. Boxplots show simulated individual values based on reported means and SDs. Genera with relative abundance values close to zero were omitted for clarity. *p*-values indicate overall group differences (*p* < 0.05). The circles in the box plots represent outliers. These are individual data points that fall outside the main distribution of the data shown by the box. NonDM, individuals with normoglycemia; preDM, prediabetes; T2D, type 2 diabetes.

At the genus level, several bacterial genera showed significant differences across glycemic groups (Figure 1 and Supplementary Table S3).

In preDM, the abundance of Firmicutes unclassified was significantly higher compared to NonDM (*p* = 0.014) and T2D (*p* = 0.012) groups. Conversely, *Romboutsia* was already significantly reduced in preDM compared to NonDM (*p* = 0.010) and further decreased in T2D (*p* < 0.001) was significantly reduced in preDM compared to NonDM (*p* = 0.010) and further decreased in T2D (*p* < 0.001). *Lawsonibacter* abundance was also significantly lower in T2D compared to preDM (*p* = 0.030). In contrast, Alphaproteobacteria unclassified was significantly enriched in preDM compared to NonDM (*p* = 0.004), while *Granulicatella* showed a decreasing trend in preDM that reversed in T2D (*p* = 0.027).

In T2D, *Clostridium sensu stricto* and *Romboutsia* were both significantly reduced compared to NonDM (*p* = 0.011 and *p* < 0.001, respectively), whereas *Lactobacillus* was significantly enriched (*p* = 0.002). *Escherichia/Shigella* abundance was significantly higher in T2D compared to both NonDM and preDM (*p* = 0.007 for both comparisons), although no difference was observed between NonDM and preDM. *Rothia* abundance was globally different across groups (*p* = 0.009), but post hoc comparisons did not reach significance.

### 3.4. Fecal Short-Chain Fatty Acids (SCFAs)

We further examined the fecal concentrations of short-chain fatty acids (SCFAs) across groups as a proxy for shifts in gut microbial activity (Table 3). Among the measured SCFAs, only fecal acetic acid concentrations were significantly lower in individuals living with T2D compared to both NonDM (*p* = 0.036) and those with preDM (*p* = 0.018). Fecal propionic and butyric acid levels showed a trend towards lower concentrations in people with T2D compared either to NonDM (*p* = 0.091) and preDM (*p* = 0.090), respectively; however, these differences did not reach statistical significance. Lastly, none of the branched-chain SCFAs (isobutyric, isovaleric) or valeric acid differed significantly among groups.

### 3.5. Correlation Between SCFAs and Gut Microbial Genera

**Figure 2:**
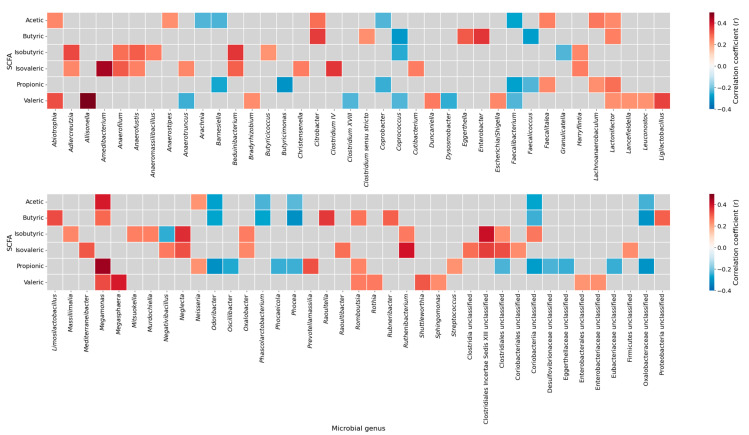
Correlation heatmap between short-chain fatty acids and gut microbial genera. Spearman correlation coefficients between SCFAs (acetic, butyric, isobutyric, isovaleric, propionic, and valeric acids) and the relative abundance of microbial genera. Positive correlations are shown in red and negative correlations in blue; gray cells indicate non-significant associations (*p* ≥ 0.05). The heatmap was generated using the complete sample set (all participants combined), without stratification by study groups. SCFAs, short-chain fatty acids.

To explore the associations between gut microbiota and fecal SCFAs, a correlation heatmap was generated (Figure 2).

The analysis identified several statistically relevant genus-level correlations with SCFAs concentrations. For instance, *Clostridium sensu stricto* showed a positive correlation with butyric acid, while *Lactonifactor* was positively associated with propionic acid. In contrast, we found that Coriobacteriia unclassified correlated negatively with both acetic and butyric acid levels. Notably, *Romboutsia* exhibited a positive correlation with propionic acid and was significantly reduced in individuals with stablished diabetes (Figure 1, Supplementary Table S3), whereas Coriobacteriia unclassified was increased in diabetes (Figure 1, Supplementary Table S3) and showed a negative correlation with acetic acid. Correlations between *Akkermansiaceae* abundance and fecal SCFA concentrations could not be estimated due to insufficient variability in this genus within our dataset (NA in correlation output). A complete list of significant correlations between individual SCFA species and bacterial genera is provided in Supplementary Table S4.

### 3.6. Microbial and Metabolic Predictors of Dysglycemia

**Figure 3:**
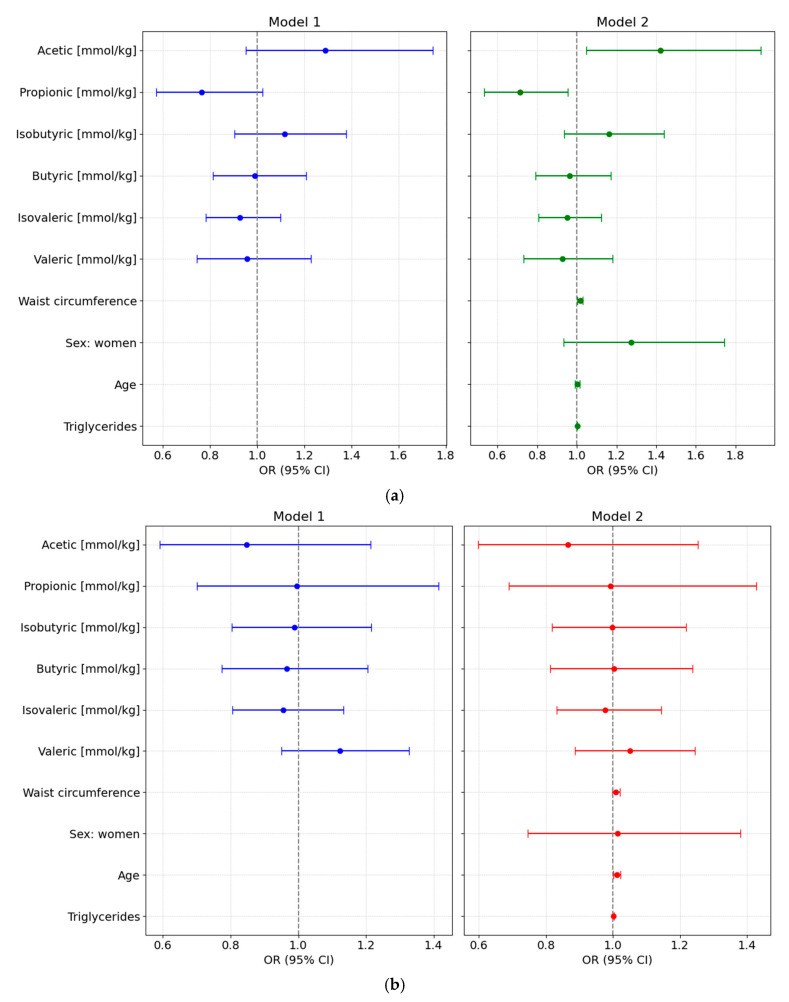

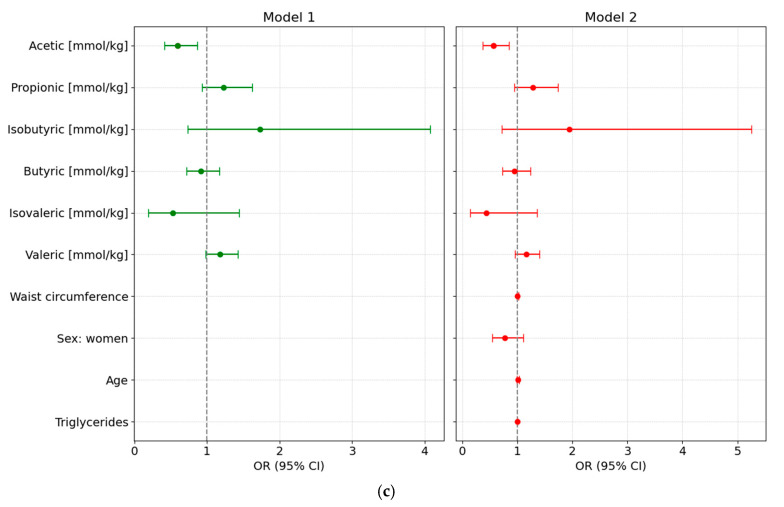
Forest plots showing odds ratios (ORs, 95% CI) from multivariable logistic regression models assessing the association of normalized fecal SCFA concentrations with glycemic status. Both unadjusted (Model 1) and adjusted models (Model 2, adjusted for waist circumference, sex, age, and triglyceride levels) are shown for each comparison: (**a**) Prediabetes vs. Normoglycemia; (**b**) Type 2 Diabetes (T2D) vs. Normoglycemia (NonDM); and (**c**) T2D vs. Prediabetes (PreDM).

We designed different adjusted multivariable logistic regression models to assess the potential of various fecal SCFA species to predict either preDM or T2D, after adjustment for clinical covariates commonly associated with altered glycemic status and metabolic risk. All main results presented below are based on models using normalized SCFA data to ensure comparability across individuals; detailed results from these normalized models are provided in Supplementary Table S5 and illustrated in Figure 3.

#### 3.6.1. PreDM vs. NonDM

Using normoglycemia as the reference category, the full-adjusted logistic regression analysis based on normalized SCFA values showed that higher levels of acetic acid were positively associated with preDM (Model 2: OR = 1.4219 [95% CI: 1.049–1.9273], *p* = 0.028), while propionic acid levels were inversely associated with this condition (OR = 0.714 [95% CI: 0.5338–0.9551], *p* = 0.028). No significant associations were observed for other SCFAs. Waist circumference was also independently associated with higher odds of preDM (OR = 1.0164 [95% CI: 1.0029–1.0301], *p* = 0.0215), whereas sex, age, and triglyceride levels were not significant predictors (Figure 3a).

#### 3.6.2. T2D vs. NonDM

In the fully adjusted multivariable model comparing T2D to NonDM, no SCFA species showed statistically significant associations. Age was the only independent variable significantly associated with higher odds of T2D, with each additional year increasing the odds by approximately 1.2% (OR = 1.012 [95% CI: 1.013–1.0228]; *p* = 0.033) (Figure 3b).

#### 3.6.3. T2D vs. PreDM

Using preDM as the reference category revealed that higher levels of acetic acid concentration were associated with lower odds of T2D (OR = 0.5605 [95% CI: 0.3713–0.8461]; *p* = 0.009). None of the other SCFA species or clinical covariates demonstrated statistically significant associations. Furthermore, none of the clinical variables, including age, were significantly associated with T2D status in this model (Figure 3c).

## 4. Discussion

An accumulating body of evidence suggests that the GM profoundly influences glucose metabolism and may contribute to glucose intolerance and T2D progression [13,27]. While most of the studies have been focused on the identification of gut microbial alterations in T2D, the understanding of specific changes in GM in subclinical stages of glucose intolerance remains largely elusive [28,29]. Additionally, the potential of using circulating or fecal SCFAs as functional readouts of microbial composition under dysglycemic conditions is still under investigation, with conflicting findings reported in the literature [30].

In the current study, we sought to address these gaps by comparing microbiota composition and SCFA concentrations across normoglycemia, prediabetes and T2D. We found a significant increase in *Escherichia*/*Shigella* abundance in subjects with T2D compared to those without T2D individuals with prediabetes, consistent with previous studies linking these taxa to increased gut permeability and metabolic endotoxemia in T2D [31,32]. Conversely, acetic-producing genera, such as *Romboutsia* and *Clostridium sensu stricto*, exhibited a consistent and significant decline from normoglycemia to prediabetes and further in T2D, supporting the hypothesis of a loss of beneficial SCFA producers associated with impaired glucose metabolism [32,33,34,35]. Interestingly, individuals with prediabetes displayed a distinct microbiota profile, with significant increases in taxa such as Firmicutes unclassified and Alphaproteobacteria unclassified compared to non-diabetic individuals. However, Firmicutes unclassified levels dropped significantly in T2D, returning to levels similar to those observed in normoglycemia. This non-linear pattern suggests that some microbiota alterations may be specific to the prediabetic state rather than reflecting a progressive shift across glycemic stages. These patterns suggest that GM shifts may precede the progression to T2D at least in part by contributing to metabolic dysregulation in subjects with prediabetes [13,36].

Consistent with these compositional changes —namely, a significant increase in *Escherichia/Shigella* and a marked reduction in acetic-producing genera such as *Romboutsia* and *Clostridium sensu stricto*—individuals with T2D showed significantly lower fecal acetic concentrations compared to both NonDM and those with preDM, while propionic and butyric concentrations were at best marginally lower, when T2D is compared with participants without diabetes and with prediabetes, respectively. Our observations align with previous studies reporting reduced acetic production in individuals with T2D, reflecting impaired fermentative capacity and dysbiosis associated with metabolic deterioration [37,38,39,40,41]. While fatty acid profiles can be influenced by both diet and host factors [11], our analysis revealed some group-specific differences in dietary intake, such as higher dairy consumption in individuals with prediabetes and increased white meat intake in those with T2D. However, these dietary differences were modest and do not fully align with the observed reductions in fecal acetic acid concentrations. This suggests that alterations in gut microbiota composition may play a more prominent role in shaping SCFA profiles than dietary patterns alone. Nevertheless, it is important to consider the Mediterranean background of all study participants, which is generally characterized by higher fiber and olive oil intake. While dietary intake was assessed in our cohort and no major differences in food group consumption were observed across glycemic status groups, the overall dietary pattern may still shape gut microbiota composition and SCFA profiles. These population-specific characteristics highlight the need for cross-population validation studies to confirm the generalizability of our findings.

Beyond their key roles in maintaining gut barrier integrity and modulating metabolic inflammation, SCFAs—especially acetic and butyric—may also target glucose metabolism [42,43,44]. Our analysis revealed that higher fecal concentrations of acetic acid were associated with a reduced risk for T2D. This observation is in line with the notion that acetic is the main fermentative product and the main circulating SCFA form in humans and rodents [45]. Consistently, oral administration of acetic acid in drinking water protected against T2D in mice without obesity [44]. Intriguingly, fecal acetic acid was linked to increased risk for prediabetes. The biological significance of this finding remains unclear, particularly given the lack of significant differences in acetic acid concentrations between normoglycemic and subjects with prediabetes. This observation should be regarded as preliminary and hypothesis-generating, and the positive association observed in the preDM model could be revealing hidden relationships not apparent in simple group comparisons even after covariate adjustment or could simply result from statistical fluctuation rather than a true biological effect.

Our analysis also showed that elevations of fecal levels of propionic acid were associated with lower odds of prediabetes, suggesting a potential protective role of this SCFA during early stages of glucose dysregulation. This observation is consistent with previous evidence indicating that propionic acid may enhance insulin sensitivity and exert anti-inflammatory effects, which together may delay or prevent metabolic deterioration [46,47].

In contrast, fecal butyric concentrations did not differ significantly across groups, and they were not predictive of prediabetes or T2D in any of the models. These findings stand in contrast to the prevailing hypothesis that the GM of subjects with T2D exhibits a decreased representation of butyric-producing taxa [33]. Furthermore, our findings also differed from those of a recent prospective study based on the same Di@bet.es cohort, which evaluated the predictive value of circulating SCFA for incident T2D over a 7-year follow-up period [48]. Notably, in that study, elevated circulating butyric levels were significantly associated with increased risk of developing T2D. These inconsistent associations across studies may, at least in part, reflect differences between the blood and fecal concentrations of specific SCFA species [49], which are likely influenced by the biological compartment assessed. In fact, whereas fecal SCFA levels may primarily reflect microbial production, plasma concentrations also capture absorption dynamics, host metabolic utilization, or compensatory physiological responses. Collectively, these findings highlight the involvement of SCFA-related pathways in glucose dysregulation, although their interpretation appears to be highly dependent on the physiological context or disease stage.

To further investigate functional relationships, genus-level correlation analyses revealed several meaningful associations. For instance, *Romboutsia* correlated positively with propionic levels and was significantly reduced in subjects with T2D. In contrast, *Escherichia/Shigella*—primarily recognized as acetic consumers [50]—were more abundant in subjects with T2D, supporting their potential role in contributing to gut dysbiosis and reduced SCFA availability. Notably, the relative abundance of *Akkermansia* did not differ significantly across glycemic groups, underscoring the functional and ecological heterogeneity of this genus, despite its frequently proposed protective role in metabolic health [51,52,53].

Taken together, our findings reinforce the hypothesis that specific compositional shifts—characterized by a loss of beneficial fermenters and an enrichment of proinflammatory or opportunistic bacteria—may directly impair the gut microbiota’s metabolic capacity to produce SCFAs. These results further suggest that interventions aimed at restoring SCFA-producing bacteria or attenuating the abundance of detrimental taxa could represent promising microbiota-targeted strategies to support metabolic health in subjects with dysglycemia.

Our study has several limitations. Most notably, the relatively small sample size restricts the generalizability of the findings. Although the limited sample size reduces the statistical power and increases the potential influence of random error, these findings provide preliminary insights and should be regarded as hypothesis-generating, pending confirmation in larger studies. Nevertheless, our results are consistent with previous reports describing similar microbial and metabolic alterations in dysglycemia [35,41], which reinforces the plausibility of our observations despite the limited sample size. Moreover, the absence of detailed information on specific classes of antidiabetic and lipid-lowering medications may represent an important confounding factor, as such treatments can modulate GM composition and fecal SCFA levels. In addition, SCFAs were quantified exclusively in fecal samples, which primarily reflect excreted intestinal microbial production and may not accurately reflect actual colonic production or systemic absorption. Another limitation of our study concerns dietary data. While intake was recorded for major food groups, the lack of detailed nutrient-level information, such as total energy, fiber, or micronutrient content, prevents a precise assessment of how these dietary factors may have influenced gut microbial composition and SCFA production. Finally, the small sample size also limited our ability to use predictive metabolic pathway tools like PICRUSt or HUMAnN2, which require larger cohorts and multi-omics data for reliable results. This limits functional interpretation of microbiota–SCFA relationships. Future research with larger datasets and integrated approaches is warranted to explore functional links between microbiota and SCFA production. Nevertheless, a key strength of this study is the inclusion of detailed dietary intake data. While some differences were observed, these were modest and unlikely to fully explain the microbial and SCFA differences. Future research should prioritize longitudinal and mechanistic approaches, ideally including both fecal and circulating SCFA measurements, detailed pharmacological data, and targeted interventions to modify gut microbiota composition or SCFA production, in order to clarify causal relationships and explore their therapeutic potential. Furthermore, in vitro and animal model studies will be interesting to clarify the specific mechanisms through which SCFAs, such as acetic and propionic acid, affect insulin sensitivity, glucose homeostasis, and inflammatory responses.

## 5. Conclusions

Our findings suggest that fecal SCFAs—particularly acetic—may serve as protective biomarkers for T2D. Unexpectedly, acetic acid was revealed to predict prediabetes. However, in this last case, the physiological relevance remains unclear. Interestingly, higher fecal levels of propionic acid were inversely associated with prediabetes, pointing to a potential protective role in early glucose dysregulation. Further longitudinal research is needed to clarify the role of SCFA concentrations as indicators of gut microbial metabolic activity and to assess their potential in predicting the development and progression to T2D over time.

## Figures and Tables

**Table 1:** Demographic, clinical, and analytical data.

Variable	NonDM (n = 39)	PreDM (n = 24)	T2D (n = 25)	*p*-Value (Global)	NonDM vs. PreDM	NonDM vs. T2D	PreDM vs. T2D
Clinical variables							
Age (y)	60.1 ± 13.8	67.4 ± 8.2	70.5 ± 9.4	<0.01	0.156	<0.01	0.907
Sex, women	19 (48.7%)	11 (45.8%)	6 (24.2%)	0.124	0.823	0.047	0.108
BMI (kg/m^2^)	27.4 ± 3.7	29.2 ± 4.5	29.5 ± 4.2	0.087	0.496	0.142	1.000
Waist circumference	88.1 ± 11.8	95.9 ± 12.6	100 ± 12.6	<0.01	0.122	<0.01	0.502
Smoking habit	5 (23.8%)	5 (38.5%)	6 (42.9%)	0.465	0.362	0.234	0.816
Hypertension, n (%)	13 (33.3%)	14 (58.3%)	16 (64.0%)	0.031	0.051	0.016	0.684
Dyslipidemia, n (%)	7 (17.9%)	8 (33.3%)	14 (56.0%)	0.007	0.163	0.001	0.110
*Medication use*							
Antidiabetic drugs (ADO) (%)				<0.001			
yes	0 (0.00%)	1 (4.17%)	21 (84.0%)				
no	39 (100%)	23 (95.8%)	4 (16.0%)				
Insulin therapy (%)				0.010			
yes	0 (0.00%)	0 (0.00%)	4 (16.0%)				
no	39 (100%)	23 (100%)	21 (84.0%)				
Serum biochemistry							
Glucose (mg/dL)	96.2 ± 9.9	108.9 ± 18.6	151.2 ± 51.0	<0.0001	0.007	<0.001	<0.001
Insulin (mIU/L)	11.8 ± 8.4	12.4 ± 12.0	9.5 ± 4.8	0.486	1.000	1.000	1.000
HbA1c (%)	5.3 ± 0.3	5.8 ± 0.3	7.0 ± 0.6	<0.001	<0.001	<0.001	<0.001
Cholesterol (mg/dL)	201.0 ± 37.5	213.0 ± 36.0	181.0 ± 35.9	0.010	1.000	0.072	0.012
LDLc (mg/dL)	126.0 ± 31.8	133 ± 31.1	106 ± 27.2	0.006	1.000	0.018	0.013
HDLc (mg/dL)	54.2 ± 11.6	52.6 ± 13.6	45.3 ± 10.3	0.013	1.000	0.022	0.160
Triglycerides (mg/dL)	106 ± 50.5	139 ± 66.1	151 ± 90.0	0.024	0.054	0.055	1.000
AST	19.8 ± 5.1	18.6 ± 8.0	22.3 ± 8.6	0.604	0.868	0.854	0.601
GGT	42.3 (45.9)	36.5 (39.8)	24.0 (12.3)	0.616	0.397	0.239	0.684
Fatty liver index (FLI)	67.2 ± 25.4	78.5 ± 14.5	77.3 ± 25.4	0.515	1.000	1.000	1.000
Urinary biochemistry							
Glucose (g/L)	0.45 (1.42)	0.08 (0.05)	2.31 (6.21)	0.051	1.000	0.199	0.812
Fructose (g/L)	0.03 (0.06)	0.01 (0.02)	0.68 (2.83)	0.360	1.000	0.568	0.481
Sucrose (g/L)	0.08 (0.22)	0.06 (0.14)	1.91 (5.99)	0.134	1.000	1.000	1.000

Continuous variables are summarized as mean (SD) and categorical variables as counts with percentages. Differences across groups were evaluated with Chi-square for categorical data and with Wilcoxon or Kruskal–Wallis tests for continuous data, implemented through the compare Groups package in R. AST, aspartate aminotransferase; BMI, body mass index; GGT, gamma-glutamyl transferase; HDLc, high-density lipoprotein cholesterol; LDLc, low-density lipoprotein cholesterol; NonDM, individuals with normoglycemia; preDM, prediabetes; T2D, type 2 diabetes.

**Table 2:** Dietary variables showing significant differences between glycemic groups.

Food Group	NonDM n = 39	PreDM n = 24	T2D n = 25	*p*-All	NonDM vs. PreDM	NonDM vs. T2D	PreDM vs. T2D
Dairy	42.7 ± 31.4	57.1 ± 28.2	49.2 ± 20.5	0.079	0.040	0.154	0.347
White meat	13.4 ± 7.1	12.7 ± 7.5	24.3 ± 22.7	0.060	0.614	0.055	0.027
Beer	7.3 ± 11.8	4.7 ± 10.2	4.1 ± 7.2	0.047	0.019	0.177	0.237
Coffee	42.3 ± 32.4	63.3 ± 31.0	48.0 ± 21.4	0.021	0.010	0.269	0.056

Data are expressed as consumption frequency (times per month). Values are presented as mean ± standard deviation (SD). NonDM, individuals with normoglycemia; preDM, prediabetes; T2D, Type 2 diabetes.

**Table 3:** Fecal SCFA concentrations (mmol/kg) by diagnostic group.

SCFA (mmol/kg)	NonDM (n = 39)	PreDM (n = 24)	T2D (n = 25)	*p*-Value (Overall)	NonDM vs. preDM	NonDM vs. T2D	PreDM vs. T2D
Acetic	173 ± 121	179 ± 109	115 ± 64.6	0.061	0.853	**0.036**	**0.018**
Propionic	52.5 ± 35.9	45.0 ± 31.1	38.1 ± 24.8	0.230	0.433	0.091	0.409
Isobutyric	5.58 ± 3.44	5.54 ± 2.80	5.34 ± 2.96	0.954	0.956	0.774	0.817
Butyric	42.7 ± 30.3	43.5 ± 27.8	31.5 ± 19.2	0.206	0.914	0.112	0.090
Isovaleric	9.16 ± 7.85	8.00 ± 4.44	8.28 ± 5.09	0.772	0.539	0.628	0.842
Valeric	7.61 ± 4.37	6.96 ± 2.26	7.72 ± 5.69	0.826	0.536	0.934	0.572

Data are presented as mean (standard deviation, SD) in mmol/kg feces. Overall and pairwise *p*-values were calculated using the Kruskal–Wallis test, with post hoc pairwise comparisons adjusted for multiple testing. Bolded *p*-values indicate statistical significance (*p* < 0.05). NonDM, Normoglycemic individuals; preDM, prediabetes; T2D, type 2 diabetes.

## Data Availability

The data presented in this study are available on request from the corresponding author.

## Supplementary Materials

The following supporting information can be downloaded at: https://www.mdpi.com/article/10.3390/nu17183003/s1. Table S1: Dietary intake characteristics by glycemic status groups. Table S2: Relative abundance of bacterial phyla across glycemic status groups. Table S3: Genus-level taxa with significant differences in relative abundance between glycemic status groups. Table S4: Significant correlations between fecal SCFA concentrations and genus-level gut microbiota. Table S5: Association between fecal SCFAs and prediabetes (vs. normoglycemia). Table S6: Association between fecal SCFAs and diabetes (vs. normoglycemia). Table S7: Association between fecal SCFAs and diabetes (vs. prediabetes). Figure S1: Relative abundance of the main bacterial phyla across glycemic groups.

## Abbreviations

The following abbreviations are used in this manuscript:

preDM	Prediabetes
T2D	Type 2 Diabetes
SCFA	Short-Chain Fatty Acids
NonDM	individuals with normoglycemia
Di@bet.es	Spanish national diabetes epidemiological study
OGTT	Oral Glucose Tolerance Test
FPG	Fasting Plasma Glucose
HbA1c	Glycated Hemoglobin
ADA	American Diabetes Association
FFQ	Food Frequency Questionnaire
GM	Gut Microbiota
MASLD	Metabolic dysfunction-associated steatotic liver disease
LDL	Low-Density Lipoprotein
HDL	High-Density Lipoprotein
AST	Aspartate Aminotransferase
GGT	Gamma-Glutamyl Transferase
FLI	Fatty Liver Index
16S rRNA	16S Ribosomal Ribonucleic Acid
OTU	Operational Taxonomic Unit
R	R statistical software
SD	Standard Deviation
CI	Confidence Interval
