# Supplementary material for: Fecal Short-Chain Fatty Acids to Predict Prediabetes and Type 2 Diabetes Risk: An Exploratory Cross-Sectional Study
Source: Nutrients. 2025 Sep 19;17(18):3003. doi: 10.3390/nu17183003 (PMC12472725; doi:10.3390/nu17183003)
Supplement: Supplementary file 1 [file nutrients-17-03003-s001.zip › nutrients-3849861-supplementary.pdf]

Fecal Short-Chain Fatty Acids to Predict Prediabetes and Type 2 Diabetes Risk: An Exploratory Cross-Sectional Study

Supplementary Methods

Fecal DNA Extraction and Sequencing for Microbiota Populations Analysis

We individually extracted total DNA from fecal samples using a QIAamp™ DNA Stool Mini Kit from QIAGEN (Hilden, Germany) from feces quantified using a Nanodrop 8000 Spectrophotometer (ThermoScientific, Waltham, MA, USA). All DNA samples were diluted to 5 ng/μL and used to amplify the V3–V4 regions of the 16S ribosomal RNA (rRNA) gene, using the following universal primers in a limited-cycle PCR:

forward primer, 5′ TCG GCA GCG TCA GAT GTG TAT AAG AGA CAG CCT ACG GGN GGC WGC AG;
reverse primer, 5′ GTC TCG TGG GCT CGG AGA TGT GTA TAA GAG ACA GGA CTA CHV GGG TAT CTA ATC C.

The above-described primers contain overhangs allowing the addition of full-length Nextera adapters with barcodes for multiplex sequencing in a second PCR step, resulting in sequencing-ready libraries with approximately 450 bp insert sizes. Libraries were eluted in a 20 μL volume and pooled for sequencing. As a control for sequencing and downstream procedures, we also used two DNA samples derived from bacterial mock communities obtained from the ZymoBIOMICSTM Microbial Community DNA Standard, a mixture of genomic DNA of ten microbial strains isolated from pure cultures of eight bacterial and two fungal strains.

Sequencing was performed on an Illumina MiSeq with 2×300 bp reads using v3 chemistry with a loading concentration of 10 pM. Sequencing was performed at the Genomics Unit of the Centre for Genomic Regulation, Barcelona.

Sequencing reads were checked for quality using FastQC. 16S amplicons were analyzed using mothur version 1.44.1 [21]. Overlapping pairs of sequence reads were assembled, contigs with more than 4 ambiguities and shorter than 439 bp or larger than 466 bp were discarded, and the remaining contigs were aligned to the reference alignment provided by the SILVA database (version 132) [22] with a k-mer size of 8. Artifacts from the alignment and the contigs with more than 12 homo-polymers (the maximum number found in the reference alignment) were removed. The resulting alignment was simplified by removing the columns containing only gaps and by discarding duplicated sequences.

The aligned sequences were then grouped, allowing up to 4 mismatches, and clusters with only one sequence were removed. Uchime (embedded in the mothur framework) was used to remove chimeras, and the resulting sequences were classified according to the taxonomy into the corresponding operational taxonomic units (OTUs). Undesired lineages such as chloroplast, mitochondria, archaea, eukaryota, and "unknown" were removed. Sequences were then clustered into OTUs using the cluster.split command at the genus level. Finally, OTUs mapping to the same genus were grouped together. The sequencing error, estimated on the mock samples, was close to zero (<1.0 × 10−10).

Supplementary Table S1. Dietary intake characteristics by glycemic status groups.

Food group	NonDM<br>N=39	preDM<br>N=24	T2D<br>N=25	*p*-All	NonDM vs preDM	NonDM vs T2D	preDM vs T2D
Vegetables	46.3 ± 32.7	51.5 ± 24.4	52.8 ± 54.4	0.260	0.133	0.582	0.217
Fruit	68.5 ± 39.2	61.9 ± 37.9	67.3 ± 66.4	0.480	0.491	0.248	0.639
Dairy	42.7 ± 31.4	57.1 ± 28.2	49.2 ± 20.5	0.079	0.040	0.154	0.347
Potatoes	12.8 ± 11.4	14.5 ± 9.3	13.9 ± 10.7	0.603	0.332	0.568	0.708
Legumes	8.9 ± 6.0	10.7 ± 7.0	8.5 ± 6.2	0.339	0.274	0.671	0.148
Nuts	10.7 ± 18.1	9.6 ± 12.2	7.3 ± 9.6	0.784	0.615	0.538	0.869
Non-refined cereals	17.3 ± 27.6	11.7 ± 19.2	5.7 ± 14.1	0.168	0.426	0.060	0.354
Fish	10.7 ± 7.4	11.6 ± 6.3	17.6 ± 30.0	0.669	0.377	0.727	0.733
Red meat	25.1 ± 15.3	22.0 ± 11.9	24.3 ± 22.7	0.471	0.539	0.272	0.421
White meat	13.4 ± 7.1	12.7 ± 7.5	24.3 ± 22.7	0.060	0.614	0.055	0.027
Wine	12.6 ± 20.7	12.6 ± 18.8	17.4 ± 22.1	0.591	0.982	0.347	0.409
Beer	7.3 ± 11.8	4.7 ± 10.2	4.1 ± 7.2	0.047	0.019	0.177	0.237
Coffee	42.3 ± 32.4	63.3 ± 31.0	48.0 ± 21.4	0.021	0.010	0.269	0.056
Sweetener	9.4 ± 17.5	6.9 ± 13.3	12.6 ± 41.9	0.566	0.499	0.319	0.753

Data are expressed as consumption frequency (times per month). Values are presented as mean ± standard deviation (SD).

NonDM, individuals with normoglycemia; preDM, prediabetes; T2D, Type 2 diabetes.

Supplementary Table S2. Relative abundance of bacterial phyla across glycemic status groups

Phyla	NonDM<br>N=39	preDM<br>N=24	T2D<br>N=25	*p*-All	NonDM vs preDM	NonDM vs T2D	preDM vs T2D
Actinobacteria	5.00 ± 4.61	4.95 ± 6.48	4.48 ± 3.82	0.912	1.000	1.000	1.000
Bacteroidetes	8.76 ± 12.70	13.5 ± 17.50	9.06 ± 9.64	0.356	0.769	1.000	1.000
Campilobacterota	0.00 ± 0.00	0.00 ± 0.00	0.00 ± 0.00	0.990	1.000	1.000	1.000
Candidatus Saccharibacteria	0.03 ± 0.03	0.03 ± 0.03	0.04 ± 0.08	0.405	1.000	1.000	1.000
Firmicutes	78.0 ± 15.50	72.4 ± 16.70	74.8 ± 17.40	0.406	0.445	1.000	1.000
Fusobacteria	0.03 ± 0.17	0.00 ± 0.00	0.02 ± 0.05	0.560	1.000	0.919	0.255
Lentisphaerae	0.01 ± 0.06	0.02 ± 0.06	0.01 ± 0.02	0.655	0.192	1.000	0.919
Proteobacteria	4.61 ± 10.6	4.94 ± 9.87	6.81 ± 11.50	0.708	1.000	0.175	1.000
Synergistetes	0.01 ± 0.03	0.01 ± 0.04	0.06 ± 0.20	0.139	1.000	0.199	0.804
Tenericutes	0.03 ± 0.19	0.15 ± 0.64	0.01 ± 0.02	0.307	1.000	1.000	1.000
Verrucomicrobia	3.52 ± 7.05	4.01 ± 6.14	4.68 ± 9.45	0.838	0.712	1.000	1.000

Values are presented as mean ± standard deviation (SD).

NonDM, individuals with normoglycemia; preDM, prediabetes; T2D, Type 2 diabetes.

Supplementary Table S3. Genus-level taxa with significant differences in relative abundance between glycaemic status groups

Bacterias Taxonomy (Genus)	NonDM N=39	preDM N=24	T2D N=25	*p*-All	NonDM vs PreDM	NonDM vs T2D	PreDM vs T2D
Rothia	0.06 ± 0.10	0.05 ± 0.10	0.23 ± 0.42	0.009	1.000	1.000	0.508
Coriobacteriia unclassified	0.02 ± 0.03	0.07 ± 0.10	0.06 ± 0.08	0.016	1.000	1.000	1.000
Barnesiella	0.20 ± 0.29	0.49 ± 0.75	0.12 ± 0.18	0.010	1.000	1.000	0.362
Clostridium sensu stricto	0.98 ± 1.85	0.42 ± 0.84	0.16 ± 0.27	0.046	0.254	0.011	0.919
Lactonifactor	0.00 ± 0.00	0.00 ± 0.00	0.00 ± 0.00	0.019	0.225	0.190	1.000
Romboutsia	0.25 ± 0.38	0.07 ± 0.15	0.04 ± 0.07	0.006	0.010	<0.001	0.769
Lawsonibacter	0.04 ± 0.04	0.05 ± 0.05	0.02 ± 0.03	0.028	1.000	0.107	0.030
Syntrophomonadaceae unclassified	0.00 ± 0.00	0.00 ± 0.00	0.00 ± 0.00	0.004	0.014	1.000	0.011
Firmicutes unclassified	2.76 ± 3.10	6.01 ± 5.19	2.57 ± 2.94	0.002	0.014	1.000	0.012
Enteroscipio	0.00 ± 0.00	0.00 ± 0.00	0.00 ± 0.00	0.057	0.195	0.046	1.000
Granulicatella	0.06 ± 0.09	0.04 ± 0.10	0.12 ± 0.24	0.167	0.072	1.000	0.027
Lactobacillus	0.03 ± 0.17	0.04 ± 0.08	0.32 ± 1.28	0.207	0.748	0.002	0.150
Limosilactobacillus	0.05 ± 0.16	0.39 ± 1.80	0.06 ± 0.18	0.329	1.000	0.040	0.457
Peptococcus	0.00 ± 0.01	0.00 ± 0.00	0.00 ± 0.00	0.527	0.042	1.000	0.059
Peptostreptococcaceae unclassified	0.10 ± 0.23	0.10 ± 0.21	0.02 ± 0.04	0.226	0.647	0.029	0.695
Butyricicoccus	0.00 ± 0.01	0.01 ± 0.02	0.00 ± 0.00	0.141	0.716	0.408	0.048
Faecalicoccus	0.01 ± 0.02	0.02 ±0.05)	0.01 ± 0.04	0.425	0.551	0.505	0.043
Alphaproteobacteria unclassified	0.05 ± 0.22	0.20 ±0.49	0.12 ± 0.34	0.283	0.004	0.553	0.258
Oxalobacteraceae unclassified	0.01 ± 0.02	0.02 ±0.02	0.01 ± 0.02	0.163	0.014	0.059	1.000
Escherichia/Shigella	2.98 ± 8.05	3.07 ± 9.80	3.55 ± 5.51	0.960	1.000	0.007	0.007

Values are presented as mean ± standard deviation (SD) of relative abundance (%) at the genus level.

NonDM, individuals with normoglycemia; preDM, prediabetes; T2D, Type 2 diabetes.

Supplementary Table S4. Significant correlations between fecal SCFA concentrations and genus-level gut microbiota

Genus	Correlation	
	r	P
**Correlation of acetic acid**		
Arachnia	-0.234	3.79e-2
Coriobacteriia unclassified	-0.267	1.72e-2
Barnesiella	-0.236	3.60e-2
Coprobacter	-0.234	3.79e-2
Odoribacter	-0.273	1.48e-2
Faecalibacterium	-0.263	1.90e-2
Phocea	-0.224	4.77e-2
Phascolarctobacterium	-0.235	3.70e-2
Oxalobacteraceae unclassified	-0.241	3.26e-2
Abiotrophia	0.242	3.15e-2
Anaerostipes	0.232	3.93e-2
Lachnoanaerobaculum	0.265	1.85e-2
Lactonifactor	0.238	3.45e-2
Faecalitalea	0.257	2.21e-2
Megamonas	0.387	4.23e-4
Neisseria	0.224	4.74e-2
Citrobacter	0.276	1.37e-2
**Correlation of propionic acid**		
Coriobacteriia unclassified	-0.281	1.22e-2
Eggerthellaceae unclassified	-0.247	2.83e-2
Phocaeicola	-0.247	2.82e-2
Barnesiella	-0.261	2.01e-2
Coprobacter	-0.247	2.83e-2
Butyricimonas	-0.297	7.92e-3
Odoribacter	-0.303	6.65e-3
Clostridiales unclassified	-0.236	3.63e-2
Eubacteriaceae unclassified	-0.247	2.80e-2
Faecalibacterium	-0.270	1.61e-2
Oscillibacter	-0.256	2.26e-2
Phocea	-0.242	3.20e-2
Faecalicoccus	-0.233	3.86e-2
Oxalobacteraceae unclassified	-0.297	7.85e-3
Desulfovibrionaceae unclassified	-0.238	3.44e-2
Prevotellamassilia	0.317	4.48e-3
Streptococcus	0.228	4.34e-2
Lachnoanaerobaculum	0.232	4.00e-2
Lactonifactor	0.280	1.23e-2
Romboutsia	0.248	2.74e-2
Faecalitalea	0.238	3.43e-2
Megamonas	0.454	2.66e-5
Neisseria	0.228	4.36e-2
**Correlation of isobutyric acid**		
Granulicatella	-0.229	4.23e-2
Coprococcus	-0.255	2.35e-2
Negativibacillus	-0.249	2.71e-2

Coriobacteriia unclassified	0.267	1.73e-2
Adlercreutzia	0.328	3.16e-3
Beduinibacterium	0.349	1.64e-3
Clostridiales Incertae Sedis XIII unclassified	0.407	1.94e-4
Clostridiales unclassified	0.234	3.78e-2
Anaerofustis	0.301	7.12e-3
Murdochiella	0.255	2.36e-2
Anaerofilum	0.277	1.35e-2
Anaeromassilibacillus	0.258	2.15e-2
Butyricicoccus	0.225	4.65e-2
Harryflintia	0.243	3.11e-2
Massiliimalia	0.245	2.92e-2
Neglecta	0.365	9.55e-4
Ruthenibacterium	0.262	1.95e-2
Mitsuokella	0.255	2.32e-2
Oxalobacter	0.259	2.13e-2
**Correlation of butyric acid**		
Coriobacteriia unclassified	-0.244	3.01e-2
Odoribacter	-0.266	1.77e-2
Coprococcus	-0.290	9.57e-3
Phocea	-0.307	5.96e-3
Faecalicoccus	-0.278	1.32e-2
Phascolarctobacterium	-0.264	1.88e-2
Oxalobacteraceae unclassified	-0.295	8.37e-3
Eggerthella	0.304	6.45e-3
Rubneribacter	0.299	7.34e-3
Limosilactobacillus	0.326	3.41e-3
Clostridium sensu stricto	0.231	4.10e-2
Lactonifactor	0.256	2.27e-2
Romboutsia	0.270	1.62e-2
Megamonas	0.287	1.04e-2
Citrobacter	0.344	1.89e-3
Enterobacter	0.354	1.35e-3
Raoultella	0.347	1.74e-3
Proteobacteria unclassified	0.299	7.44e-3
**Correlation of isovaleric acid**		
Cutibacterium	0.258	2.15e-2
Coriobacteriales unclassified	0.235	3.70e-2
Adlercreutzia	0.245	2.93e-2
Raoultibacter	0.277	1.36e-2
Clostridia unclassified	0.282	1.18e-2
Beduinibacterium	0.298	7.68e-3
Christensenella	0.260	2.06e-2
Clostridiales Incertae Sedis XIII unclassified	0.341	2.12e-3
Clostridiales unclassified	0.323	3.74e-3
Anaerofustis	0.252	2.52e-2
Mediterraneibacter	0.310	5.37e-3
Anaerofilum	0.304	6.48e-3
Anaerotruncus	0.241	3.23e-2
Clostridium IV	0.349	1.60e-3
Harryflintia	0.258	2.15e-2

Negativibacillus	0.263	1.90e-2
Neglecta	0.315	4.64e-3
Ruthenibacterium	0.395	3.19e-4
Amedibacterium	0.447	3.69e-5
Firmicutes unclassified	0.236	3.62e-2
Oxalobacter	0.245	2.97e-2
**Correlation of valeric acid**		
Coprococcus	-0.231	4.01e-2
Anaerotruncus	-0.242	3.17e-2
Dysosmobacter	-0.251	2.60e-2
Faecalibacterium	-0.228	4.36e-2
Clostridium XVIII	-0.230	4.16e-2
Rothia	0.263	1.91e-2
Lancefieldella	0.226	4.50e-2
Duncaniella	0.262	1.96e-2
Abiotrophia	0.321	3.91e-3
Leuconostoc	0.227	4.42e-2
Ligilactobacillus	0.336	2.45e-3
Lactonifactor	0.232	3.96e-2
Shuttleworthia	0.307	5.91e-3
Romboutsia	0.260	2.05e-2
Megamonas	0.327	3.23e-3
Allisonella	0.492	4.04e-6
Megasphaera	0.391	3.67e-4
Bradyrhizobium	0.233	3.84e-2
Sphingomonas	0.233	3.88e-2
Enterobacterales unclassified	0.224	4.70e-2
Enterobacteriaceae unclassified	0.231	4.09e-2
Escherichia/Shigella	0.246	2.88e-2

Spearman correlation coefficients (r) and associated p-values showing significant associations between fecal short-chain fatty acid (SCFA) species and bacterial genera. Only correlations with $p < 0.05$ are reported.

SCFA, short-chain fatty acid.

Supplementary Table S5. Association Between Fecal SCFAs and Prediabetes (vs. Normoglycemia).

Variable	Model 1 OR (95% CI)	p-value	Model 2 OR (95% CI)	p-value
Acetic acid (mmol/kg)	1.289 (0.952 – 1.745)	0.108	1.422 (1.049 – 1.927)	0.028*
Propionic acid (mmol/kg)	0.765 (0.572 – 1.022)	0.076	0.714 (0.534 – 0.955)	0.028*
Isobutyric acid (mmol/kg)	1.116 (0.904 – 1.378)	0.314	1.160 (0.937 – 1.439)	0.180
Butyric acid (mmol/kg)	0.989 (0.811 – 1.207)	0.916	0.963 (0.791 – 1.171)	0.705
Isovaleric acid (mmol/kg)	0.926 (0.781 – 1.098)	0.383	0.952 (0.808 – 1.123)	0.565
Valeric acid (mmol/kg)	0.955 (0.744 – 1.227)	0.723	0.928 (0.731 – 1.179)	0.546
Waist circumference (cm)			1.016 (1.003 – 1.030)	0.022*
Sex, women			1.213 (0.932 – 1.744)	0.136
Age (years)			1.003 (0.992 – 1.015)	0.535
Triglycerides (mg/dL)			1.002 (1.000 – 1.004)	0.141

Multivariable logistic regression models using z-score normalized SCFAs. Significant associations are marked with (*).

Supplementary Table S6. Association Between Fecal SCFAs and Diabetes (vs. Normoglycemia)

Variable	Model 1 OR (95% CI)	p-value	Model 2 OR (95% CI)	p-value
Acetic acid (mmol/kg)	0.847 (0.590 – 1.215)	0.370	0.865 (0.597 – 1.254)	0.045
Propionic acid (mmol/kg)	0.996 (0.701 – 1.414)	0.981	0.993 (0.689 – 1.429)	0.968
Isobutyric acid (mmol/kg)	0.988 (0.803 – 1.216)	0.909	0.999 (0.818 – 1.219)	0.989
Butyric acid (mmol/kg)	0.965 (0.774 – 1.205)	0.756	1.004 (0.814 – 1.238)	0.973
Isovaleric acid (mmol/kg)	0.955 (0.806 – 1.133)	0.602	0.976 (0.833 – 1.145)	0.770
Valeric acid (mmol/kg)	0.123 (0.950 – 1.328)	0.180	1.051 (0.886 – 1.246)	0.573
Waist circumference (cm)			1.009 (0.997 – 1.021)	0.140
Sex, women			1.015 (0.745 – 1.381)	0.927
Age (years)			1.012 (1.001 – 1.023)	0.033*
Triglycerides (mg/dL)			1.001 (0.999 – 1.003)	0.307

Multivariable logistic regression models using z-score normalized SCFAs. Significant associations are marked with (*).

Supplementary Table S7. Association Between Fecal SCFAs and Diabetes (vs. Prediabetes)

Variable	Model 1 OR (95% CI)	p-value	Model 2 OR (95% CI)	p-value
Acetic acid (mmol/kg)	0.598 (0.412 – 0.869)	0.010*	0.561 (0.371 – 0.846)	0.009*
Propionic acid (mmol/kg)	1.232 (0.935 – 1.623)	0.146	1.281 (0.945 – 1.738)	0.120
Isobutyric acid (mmol/kg)	1.735 (0.739 – 4.073)	0.214	1.947 (0.721 – 5.259)	0.198
Butyric acid (mmol/kg)	0.918 (0.721 – 1.170)	0.494	0.944 (0.724 – 1.231)	0.673
Isovaleric acid (mmol/kg)	0.526 (0.192 – 1.444)	0.220	0.431 (0.137 – 1.358)	0.159

Valeric acid (mmol/kg)	1.181 (0.981 – 1.423)	0.0869	1.156 (0.951 – 1.405)	0.155
Waist circumference (cm)			1.001 (0.987 – 1.015)	0.903
Sex, women			0.772 (0.547 – 1.103)	0.165
Age (years)			1.009 (0.990 – 1.028)	0.377
Triglycerides (mg/dL)			0.999 (0.997 – 1.001)	0.512

Multivariable logistic regression models using z-score normalized SCFAs. Significant associations are marked with (*).

Supplementary Figure S1. Relative abundance of the main bacterial phyla across glycemic groups

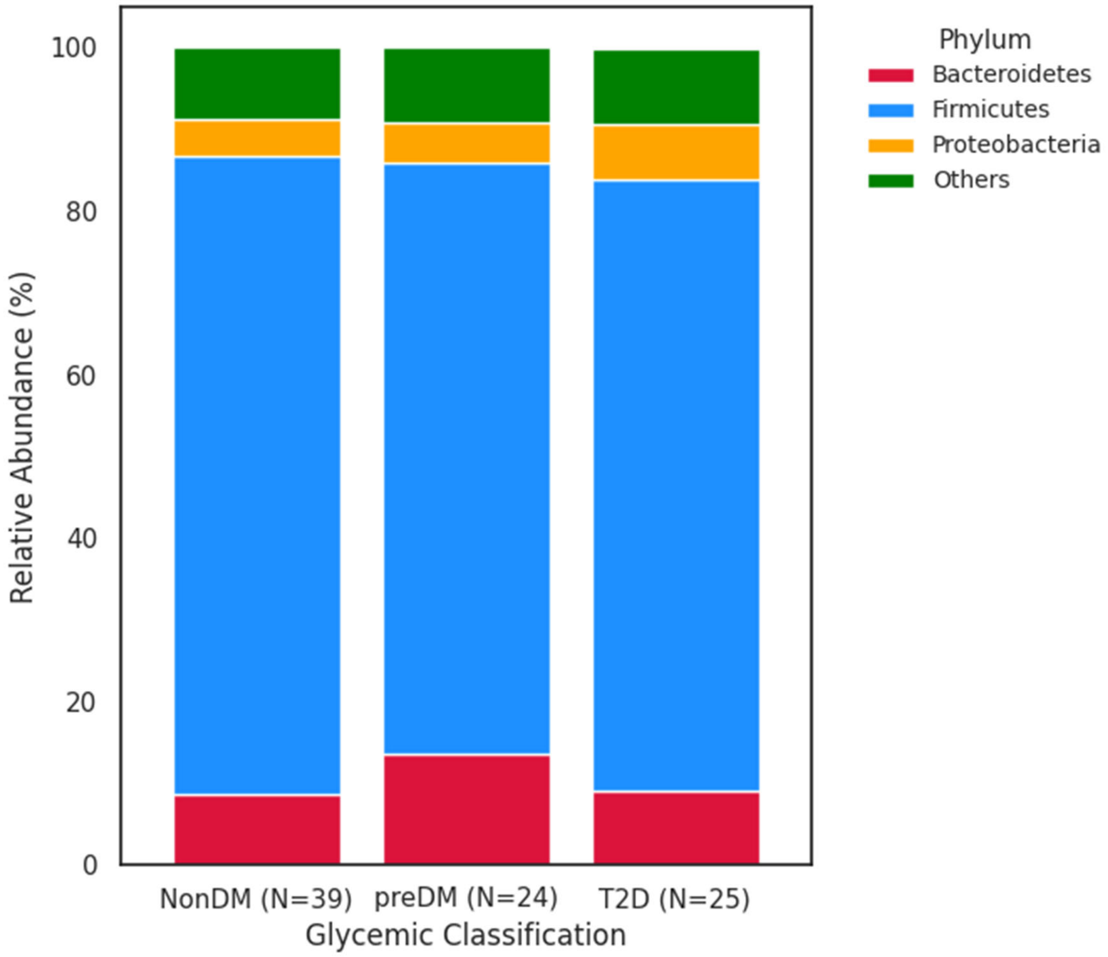

NonDM, individuals with normoglycemia; preDM, prediabetes; T2D, Type 2 diabetes.
